# Cardiac autonomic dysfunction and structural remodeling: the potential mechanism to mediate the relationship between obstructive sleep apnea and cardiac arrhythmias

**DOI:** 10.3389/fmed.2024.1346400

**Published:** 2024-04-02

**Authors:** Hao Chen, Qingfeng Zhang, Yueying Hao, Jingyi Zhang, Yang He, Ke Hu

**Affiliations:** Department of Respiratory and Critical Care Medicine, Renmin Hospital of Wuhan University, Wuhan, China

**Keywords:** sleep disordered breathing, obstructive sleep apnea, cardiac arrhythmias, atrial fibrillation, non-sustained ventricular tachycardia, heart rate variability

## Abstract

**Background:**

Cardiac arrhythmias are very common in patients with obstructive sleep apnea (OSA), especially atrial fibrillation (AF) and nonsustained ventricular tachycardia (NVST). Cardiac autonomic dysfunction and structural remodeling caused by OSA provide the milieu for cardiac arrhythmia development. This study aimed to determine whether OSA is associated with various cardiac arrhythmias and investigate potential pathophysiologic pathways between them.

**Methods:**

The analysis covered 600 patients with clinical suspicion of OSA hospitalized in Renmin Hospital of Wuhan University between January 2020 and May 2023. After undergoing sleep apnea monitor, all subjects received laboratory tests, Holter electrocardiography, and Echocardiography.

**Results:**

Compared with those without OSA and adjusting for potential confounders, subjects with moderate OSA had three times the odds of AF (odds ratio [OR] 3.055; 95% confidence interval [CI], 1.002–9.316; *p* = 0.048). Subjects with severe OSA had three times the odds of AF (OR 3.881; 95% CI, 1.306–11.534; *p* = 0.015) and NSVT (OR 3.690; 95% CI, 0.809–16.036; *p* = 0.046). There were significant linear trends for the association between OSA severity with AF and NVST (*p* < 0.05). And this association was mediated by cardiac structural changes including left atrial diameter, left ventricular diastolic diameter, right atrial diameter and right ventricular diameter. In addition, the ratio of low-frequency and high-frequency individually mediated the association between severe OSA and NVST.

**Conclusion:**

This study demonstrated that severe OSA was independently associated with AF and NSVT, and this association was mediated by autonomic nervous system changes and cardiac structural remodeling.

## Introduction

Sleep disordered breathing (SDB) mainly includes two types: central sleep apnea (CSA) and obstructive sleep apnea (OSA). OSA is a more common and increasingly recognized complex disorder. It is characterized by the occurrence of upper airway collapse, resulting in disruptive snoring, intermittent hypoxemia, sleep fragmentation, worsening sleep quality and excessive daytime sleepiness (1). According to estimates, the overall prevalence of OSA in the general population ranges from about 9–38% (2). Moreover, OSA is an independent risk factor for several diseases, such as hypertension (3, 4), diabetes (5), coronary artery disease (6), and cardiac arrhythmias (1, 7). Previous studies have demonstrated that OSA significantly increases the risk of several cardiac arrhythmias, including atrial fibrillation (AF), nonsustained ventricular tachycardia (NVST), and conduction delay arrhythmias (8–11).

However, few studies have analyzed the causes of arrhythmias in OSA in population studies. Several mechanisms, including cardiac autonomic dysfunction and structural remodeling caused by OSA, might explain these results (1, 3, 12). Our aim was to determine whether OSA is associated with various cardiac arrhythmias and investigate potential pathophysiologic pathways between them.

## Methods

### Study group

**Figure 1:**
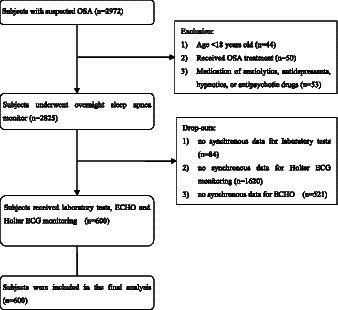
Flow diagram of this study. OSA, obstructive sleep apnea; ECG, electrocardiography; ECHO, echocardiography.

Figure 1 depicts a flowchart illustrating the study design and process. To ensure the validity of the assessment, the exclusion criteria were: age <18 years; ongoing OSA therapy; current use of anxiolytics, antidepressants, hypnotics, or antipsychotic drugs.

From January 2020 to May 2023, a total of 2,972 subjects were initially recruited. Among them, 147 subjects were excluded from formal enrollment due to age <18 years (*n* = 44), received OSA treatment (*n* = 50) or medication of anxiolytics, antidepressants, hypnotics, or antipsychotic drugs (*n* = 53). After undergoing overnight sleep apnea monitor, 600 subjects received laboratory tests, Echocardiography (ECHO) and Holter electrocardiography monitoring. Finally, this retrospective observational study included 600 subjects with clinical suspicion of OSA in Renmin Hospital of Wuhan University.

From the electronic medical records, we collected essential clinical information, including demographic and anthropometric data (such as sex, age, height, weight, systolic blood pressure, and diastolic blood pressure), as well as details related to cardiovascular risk factors (such as smoking, drinking, and diabetes) and cardiovascular diseases (such as hypertension, coronary heart disease, heart failure, and myocardial infarction).

All enrolled subjects signed written informed consent. The study has been approved by the ethics committee of Renmin Hospital of Wuhan University. All studies have been conducted according to the ethics guidelines laid down in the Helsinki Declaration.

### Biochemical measurements

All subjects had undergone fasting blood tests including blood glucose, low-density lipoprotein cholesterol (LDL), high-density lipoprotein cholesterol (HDL), triglyceride (TG), total cholesterol (TC), brain natriuretic peptide (BNP), Creatinine (Cr), K, Na, CI, Ca, Activated Partial Prothrombin Time (APPT), Prothrombin Time (PT), Fibrinogen and D-Dimer.

### Holter electrocardiography and heart rate variability analysis

HRV measurement was used to assess cardiac autonomic dysfunction. We analyzed the frequency and time domain of HRV by using Holter electrocardiography monitoring (BI9800, Biomedical Instruments Co., Ltd., Shenzhen, Guangdong, China). The time domain analysis includes parameters such as: standard deviation of normal-to-normal intervals (SDNN); standard deviation of 5 min average NN intervals (SDNN Index); standard deviation of 5 min average NN intervals (SDANN); root mean square successive difference (RMSSD); percentage of adjacent NN intervals (PNN50); and trigonometric index (11). The frequency analysis includes parameters such as: power in high frequency spectrum (HF; 0.15–0.4 Hz), power in low frequency spectrum (LF; 0.04–0.15 Hz), power in very low frequency spectrum (VLF; 0.003–0.04 Hz) and the ratio of the two values (LF/HF) (12).

RMSSD and HF predominantly represent cardiac parasympathetic nerve activity, whereas LF is associated with cardiac sympathetic nerve activity. Consequently, the LF/HF ratio serves as an indicator of the balance between sympathetic and parasympathetic functions, with elevated LF/HF values suggesting heightened sympathetic nerve excitability (11, 12).

### Echocardiography

All subjects received transthoracic echocardiography with Color Doppler Echocardiography (ECHO) performed by an experienced ultrasound physician. The ascending aortic diameter (AAOD), main pulmonary artery diameter (MPAD), left atrial diameter (LAD), left ventricular diastolic diameter (LVDD), right atrial diameter (RAD), right ventricular diameter (RVD), left ventricular systolic diameter (LVSD) and left ventricular posterior wall thickness (LVPWD) were measured using standard M-mode ECHO. The aortic valve stroke volume (AVSV), mitral valve e peak velocity (MVE) and left ventricular ejection fraction (LVEF) values of each patient were obtained from the results of ECHO.

### Sleep apnea parameters

Each subject was monitored for at least 7 h of sleep using a portable and non-contact sleep apnea monitor (Megasens Technology Co. Ltd., Hangzhou, China), including an ultra-wideband sleep apnea monitor (OrbSense, ZG-S01D) and blood oxygen saturation monitor (ZG-P11F). All of the subjects underwent 7 hours nighttime monitoring with this device in bed. No ingest caffeine, nap or engage and protracted or tedious exercises, and so on were informed during the study. The study recorded the following parameters: (1) Apnea-Hypopnea Index (AHI), which is the average number of apnea and hypopnea episodes per hour of sleep; (2) An apnea, which is a reduction in nasal airflow of at least ≥10 s 80%; (3) A hypopnea, which is a decrease in nasal airflow ≥30% and a decrease in oxygen saturation ≥4%; (4) the lowest and mean arterial oxygen saturation (LSaO2 and MSaO2), estimated by pulse oximetry during sleep [%], (5) Oxygen Desaturation Index (ODI), which is the number of average hourly decreases in oxygen saturation of ≥4%; and (6) the total sleep time with oxygen saturation below 90% (TST90) [%] (8–10). Subjects were categorized into four groups based on AHI by using common clinical cutoff points: none (AHI < 5), mild (5 ≤ AHI < 15), moderate (15 ≤ AHI < 30), and severe (AHI ≥ 30).

### Statistical analysis

Based on the specific distribution and type of data, the results are presented as either mean ± standard deviation or number (percentage). Analysis of variance (ANOVA) was used for the normal distribution of data, and the Welch’s test was used for non-normally distributed data. The chi-squared test was used to compare categorical data. The relationship between variables was tested by Pearson and Spearman correlations, and multiple linear regression analyses were performed to evaluate the associations between dependent and independent variables. Statistical analyses were performed using SPSS v26.0 (IBM Corp., Armonk, NY, United States) and R program (v4.0; R Foundation, Vienna, Austria). The significance level of *p* < 0.05 was considered statistically significant for the entire analysis.

Multivariate logistic regression analysis was conducted to test for the correlation between OSA severity with AF and NSVT. Multivariate linear regression analysis was utilized in determining associations between OSA severity and various measurements of HRV and ECHO. Age, sex, BMI, drinking, smoking, hypertension, diabetes and myocardial infarction were considered as covariates in the multivariable models. In this study, mediation analysis was utilized for test whether the correlations of severe OSA with AF and NVST were mediated by HRV and ECHO indicators.

## Results

### Baseline characteristics

From January 2020 to May 2023, after screening 2,972 subjects with clinical suspicion of OSA in Renmin Hospital of Wuhan University, 600 subjects fulfilled these inclusions and were included in this study (Figure 1).

Table 1 summarizes the baseline characteristics of the study population and the four subgroups based on OSA severity. Of 600 subjects, 522 (87.0%) were diagnosed with OSA, including 145 with mild, 157 with moderate, and 122 with severe OSA. As expected, OSA severity was found to be associated with BMI and male gender. The incidence of smoking, drinking, hypertension, diabetes mellitus and myocardial infarction tended to increase in subjects with more severe OSA. The distribution of coronary artery disease and heart failure did not vary significantly among patients with and without sleep apnea. AAOD, MPAD, LAD, LVDD, RAD, RVD, BNP, Cr and fasting glucose were significantly higher in subjects with OSA than in those without OSA (all *p* < 0.05). In contrast, subjects with OSA had a significantly lower LVEF in comparison to those without OSA (*p* < 0.05). Table 1 also summarizes the sleep study parameters.

**Table 1:** Characteristics of subjects with different OSA severity.

Variables	None OSA (*n* = 78)	Mild OSA (*n* = 145)	Moderate OSA (*n* = 157)	Severe OSA (*n* = 220)	*p*-value
Ages (years)	59.8 ± 14.3	61.9 ± 13.7	61.9 ± 12.4	60.9 ± 14.3	0.649
Gender (male, %)	52 (66.7%)	99 (68.3%)	110 (70.1%)	183 (83.2%)	0.001
BMI (kg/m^2^)	24.8 ± 3.7	24.9 ± 4.2	25.4 ± 4.2	27.4 ± 5.4	<0.001
SBP (mmHg)	132.4 ± 22.5	135.5 ± 19.7	132.4 ± 23.9	134.2 ± 23.1	0.412
DBP (mmHg)	75.7 ± 14.6	80.6 ± 14.7	79.5 ± 14.8	80.1 ± 14.5	0.096
Smoking	24 (30.8%)	55 (37.9%)	66 (42.0%)	109 (49.5%)	0.018
Drinking	16 (20.5%)	48 (33.1%)	40 (25.5%)	77 (35.0%)	0.043
Comorbidities, *n* (%)					
Hypertension	47 (60.3%)	94 (64.8%)	99 (63.1%)	165 (75.0%)	0.024
Diabetes mellitus	10 (12.8%)	27 (18.6%)	37 (23.6%)	71 (32.3%)	0.001
Coronary heart disease	26 (33.3%)	51 (35.2%)	59 (37.6%)	79 (35.9%)	0.930
Heart failure	8 (10.3%)	19 (13.1%)	24 (15.3%)	44 (20.0%)	0.137
Myocardial infarction	2 (2.6%)	3 (2.1%)	5 (3.2%)	18 (8.2%)	0.019
ECHO indicators					
AAOD (mm)	32.5 ± 4.0	33.2 ± 3.8	33.2 ± 3.7	34.4 ± 3.8	<0.001
MPAD (mm)	21.5 ± 2.3	21.9 ± 2.8	21.8 ± 2.9	22.6 ± 3.4	0.019
LAD (mm)	35.1 ± 5.0	36.4 ± 6.0	36.2 ± 6.7	38.9 ± 7.1	<0.001
LVDD (mm)	45.3 ± 3.4	46.1 ± 4.9	46.3 ± 5.8	48.4 ± 7.2	0.001
RAD (mm)	34.7 ± 3.8	35.0 ± 5.5	35.5 ± 5.4	37.1 ± 5.9	<0.001
RVD (mm)	20.4 ± 2.0	21.1 ± 3.0	21.0 ± 2.9	21.8 ± 2.4	<0.001
LVSD (mm)	9.9 ± 1.4	10.1 ± 1.7	10.1 ± 1.8	10.3 ± 1.8	0.149
LVPWD (mm)	9.8 ± 1.3	9.9 ± 1.3	9.9 ± 1.6	10.5 ± 3.7	0.021
AVSV (cm/s)	133.6 ± 37.8	129.2 ± 27.0	128.1 ± 26.1	127.8 ± 26.0	0.633
MVE (cm/s)	71.7 ± 20.8	72.3 ± 23.2	73.5 ± 24.1	73.6 ± 21.5	0.764
LVEF (%)	58.6 ± 2.7	57.1 ± 7.8	56.9 ± 7.3	55.6 ± 8.8	0.024
Sleeping indicators					
AHI, events/h	2.7 ± 1.4	9.7 ± 2.8	21.9 ± 4.6	48.5 ± 12.6	<0.001
ODI, events/h	3.9 ± 3.4	11.8 ± 8.2	22.7 ± 11.8	41.3 ± 18.2	<0.001
MSaO2 (%)	96.0 ± 1.6	94.9 ± 1.5	93.5 ± 3.3	92.6 ± 3.0	<0.001
LSaO2 (%)	86.5 ± 6.2	80.9 ± 9.9	77.4 ± 9.5	70.4 ± 12.9	<0.001
TST90 (%)	0.6 ± 1.3	2.9 ± 3.7	10.6 ± 14.9	17.3 ± 15.6	<0.001
Biochemical indicators					
BNP (pg/mL)	268.9 ± 567.3	545.2 ± 1136.6	666.8 ± 1420.4	1043.9 ± 4014.6	0.002
TCH (mmol/L)	4.1 ± 1.1	4.2 ± 1.1	4.0 ± 0.9	4.1 ± 1.0	0.794
TG (mmol/L)	1.6 ± 1.1	1.7 ± 1.3	1.6 ± 0.9	1.7 ± 1.2	0.127
HDL (mmol/L)	1.1 ± 0.3	1.0 ± 0.3	1.1 ± 0.2	1.1 ± 0.3	0.112
LDL (mmol/L)	2.4 ± 1.0	2.4 ± 0.8	2.3 ± 0.8	2.4 ± 0.8	0.811
Cr (μmol/L)	70.3 ± 18.9	73.9 ± 20.1	77.2 ± 35.0	81.9 ± 37.5	0.015
K (mmol/L)	4.0 ± 0.4	3.9 ± 0.4	3.9 ± 0.3	4.0 ± 0.8	0.284
Na (mmol/L)	141.1 ± 2.2	140.7 ± 2.8	141.1 ± 2.8	141.0 ± 3.6	0.978
Cl (mmol/L)	106.6 ± 2.4	106.3 ± 3.4	106.6 ± 3.8	106.2 ± 3.0	0.227
Ca (mmol/L)	2.2 ± 0.1	2.2 ± 0.2	2.2 ± 0.1	2.2 ± 0.2	0.056
Glucose (mmol/L)	5.7 ± 3.1	5.6 ± 3.9	5.6 ± 2.3	6.1 ± 2.0	<0.001
PT (sec)	11.5 ± 3.3	11.6 ± 2.2	11.6 ± 2.2	11.5 ± 1.9	0.709
APPT (sec)	28.4 ± 3.1	27.5 ± 3.4	27.5 ± 3.9	27.6 ± 3.8	0.174
FIB (g/L)	3.2 ± 1.9	3.1 ± 1.1	3.0 ± 1.1	3.3 ± 1.3	0.154
D-Dimer (mg/L)	0.5 ± 0.6	1.0 ± 4.6	1.0 ± 2.9	0.7 ± 1.4	0.480

Data are shown as mean ± SD or as number of cases (percentage). BMI, body mass index; SBP, systolic blood pressure; DBP, diastolic blood pressure; UCG, ultrasonic cardiogram with color doppler; AAOD, ascending aortic diameter; MPAD, main pulmonary artery diameter; LAD, left atrial diameter; LVDD, left ventricular diastolic diameter; RAD, right atrial diameter; RVD, right ventricular diameter; LVSD, left ventricular systolic diameter; LVPWD, left ventricular posterior wall thickness; AVSV, aortic valve stroke volume; MVE, mitral valve E peak velocity; LVEF, left ventricular ejection fraction; AHI, apnea-hypopnea index; ODI, oxygen desaturation index; MSaO2, mean oxygen saturation; LSaO2, lowest oxygen saturation; TST90, total sleep time with oxygen saturation under 90%; BNP, brain natriuretic peptide; TCH, total cholesterol; TG, triglyceride; HDL, high-density lipoprotein; LDL, low-density lipoprotein; Cr, creatinine; PT, prothrombin time; APPT, activated partial prothrombin time; FIB, fibrinogen.

### Holter electrocardiographic and HRV data

Table 2 summarizes the types of arrhythmias in subgroups based on OSA severity. The prevalence of AF and ventricular arrhythmias increased with the worsening OSA severity. There were no significant differences between groups regarding sinus arrhythmias and conduction delay arrhythmias. In addition, as shown in Table 3, considering the impact of AF on HRV, HRV parameters were compared among the different groups that excluded atrial fibrillation. The 24 h mean heart rate (HR), LF and LF/HF increased significantly as the worsened OSA severity. In contrast, SDNN, RMSSD and PNN50 decreased significantly as the worsened OSA severity.

**Table 2:** Frequency and type of arrhythmias in different OSA groups.

Variables	None OSA (*n* = 78)	Mild OSA (*n* = 145)	Moderate OSA (*n* = 157)	Severe OSA (*n* = 220)	*p*-value
Sinus arrhythmia					
Sinus tachycardia >100 bpm	9 (11.5%)	16 (11.0%)	13 (8.3%)	25 (11.4%)	0.770
Sinus bradycardia <50 bpm	4 (5.1%)	9 (6.2%)	15 (9.6%)	19 (8.6%)	0.539
Sinus pauses >2 s	6 (7.7%)	16 (11.0%)	18 (11.5%)	31 (14.1%)	0.481
Supraventricular arrhythmias					
Isolated PAC	60 (76.9%)	109 (75.2%)	123 (78.3%)	160 (72.7%)	0.504
Paired PAC	31 (39.7%)	58 (40.0%)	57 (36.3%)	87 (39.5%)	0.901
Atrial bigeminy	11 (14.1%)	24 (16.6%)	23 (14.6%)	30 (13.6%)	0.363
Atrial trigeminy	10 (12.8%)	19 (13.1%)	19 (12.1%)	30 (13.6%)	0.978
Atrial fibrillation	4 (5.1%)	22 (15.2%)	24 (15.3%)	44 (20.0%)	0.021
Supraventricular tachycardia	19 (24.4%)	24 (16.6%)	39 (24.8%)	45 (20.5%)	0.303
Ventricular arrhythmias					
Isolated PVC	38 (48.7%)	95 (65.5%)	97 (61.8%)	155 (70.5%)	0.006
Paired PVC	5 (6.4%)	24 (16.6%)	18 (11.5%)	54 (24.5%)	<0.001
Ventricular bigeminy	5 (6.4%)	14 (9.7%)	16 (10.2%)	42 (19.1%)	0.005
Ventricular trigeminy	6 (7.7%)	13 (9.0%)	18 (11.5%)	38 (17.3%)	0.045
Non-sustained VT	2 (2.6%)	5 (3.4%)	6 (3.8%)	24 (10.9%)	0.003
Conduction delay arrhythmias					
AVB type I–III	3 (3.8%)	2 (1.4%)	3 (1.9%)	7 (3.2%)	0.579

Data are shown as number of cases (percentage). VPB, ventricular premature beat; PAC, premature atrial complex; PVC, premature ventricular complexes; VT, ventricular tachycardia; AVB, atrioventricular block.

**Table 3:** Comparison of heart rate variability parameters between different OSA groups with atrial fibrillation removal.

Variables	None OSA (*n* = 74)	Mild OSA (*n* = 123)	Moderate OSA (*n* = 133)	Severe OSA (*n* = 176)	*p*-value
Mean HR (bmp)	72.5 ± 12.4	74.0 ± 10.9	73.5 ± 11.0	75.9 ± 12.3	0.009
Maximal HR (bmp)	114.2 ± 19.9	115.8 ± 19.7	111.6 ± 15.4	115.2 ± 19.5	0.350
Minimal HR (bmp)	52.6 ± 10.5	52.6 ± 9.2	53.0 ± 9.6	53.4 ± 10.6	0.684
Mean QTc (ms)	398.9 ± 33.1	397.6 ± 35.6	396.3 ± 31.6	406.4 ± 42.1	0.333
Maximal QTc (ms)	476.1 ± 58.3	470.4 ± 54.3	475.1 ± 58.5	483.5 ± 61.7	0.541
Time domain					
SDNN (ms)	122.2 ± 39.6	110.3 ± 33.9	111.4 ± 36.1	107.9 ± 33.2	0.042
SDANN (ms)	105.1 ± 39.7	99.8 ± 31.7	97.9 ± 34.3	93.4 ± 35.4	0.371
SDNN Index (ms)	50.7 ± 25.9	45.4 ± 17.9	46.9 ± 17.8	47.9 ± 19.3	0.343
RMSSD (ms)	35.9 ± 29.5	29.7 ± 21.5	29.0 ± 15.8	27.9 ± 15.2	0.042
PNN50 (%)	12.4 ± 13.5	7.6 ± 9.4	7.4 ± 8.5	7.5 ± 8.3	0.002
Trigonometric index (ms)	28.6 ± 10.1	25.5 ± 9.9	26.2 ± 10.8	25.3 ± 10.6	0.144
Frequency domain					
LF (ms^2^)	287.8 ± 236.8	303.2 ± 256.5	349.2 ± 506.7	471.6 ± 770.8	0.034
HF (ms^2^)	251.9 ± 240.8	287.0 ± 384.6	294.1 ± 521.6	354.6 ± 786.5	0.607
VLF (ms^2^)	811.3 ± 376.2	819.3 ± 498.7	874.9 ± 513.2	1011.9 ± 610.3	0.120
LF/HF	1.6 ± 1.1	1.7 ± 1.4	1.8 ± 1.2	2.1 ± 1.9	0.031

Data are shown as mean ± SD. HR, heart rate estimated by pulse oximetry; QTc, corrected QT interval; SDNN, standard deviation of normal-to-normal intervals; SDANN, standard deviation of 5 min average NN intervals; RMSSD, root mean square successive difference; PNN50, percentage of adjacent NN intervals; LF, power in low frequency spectrum; HF, power in high frequency spectrum; VLF, power in very low frequency spectrum; LF/HF, ratio of low frequency and high frequency.

### Correlation analysis between OSA with AF and OSA with NSVT

Table 4 demonstrates that unadjusted and adjusted logistic regression examined the association between OSA with AF and NSVT. Compared with the group with none OSA, subjects with mild OSA had three times the unadjusted odds of AF (odds ratio [OR] 3.309; 95% confidence interval [CI], 1.097–9.977; *p* = 0.034). Subjects with moderate OSA had three times the unadjusted odds of AF (OR 3.338; 95% CI, 1.116–9.989; *p* = 0.031). Subjects with severe OSA had four times the unadjusted odds of AF (OR 4.624; 95% CI, 1.604–13.336; *p* = 0.005) and NSVT (OR 4.653; 95% CI, 1.073–20.169; *p* = 0.040). After further adjustments for age, gender, BMI, smoking, drinking, hypertension, diabetes mellitus and myocardial infarction, subjects with moderate OSA had three times the odds of AF (OR 3.055; 95% CI, 1.002–9.316; *p* = 0.048). Subjects with severe OSA had three times the odds of AF (OR 3.881; 95% CI, 1.306–11.534; *p* = 0.015) and NSVT (OR 3.690; 95% CI, 0.809–16.036; *p* = 0.046). In the unadjusted and adjusted model, there were significant linear trends for the association between OSA severity with AF and NVST (*P* for linear trends <0.05).

**Table 4:** Unadjusted and adjusted association between OSA (categorized by severity) and cardiac arrhythmias.

	OSA category	*n* (%)	Unadjusted adjusted			
			OR (95% CI)	*p*-value	OR (95% CI)	*p*-value
AF	None	4 (5.1%)	Reference	Reference	Reference	Reference
	Mild	22 (15.2%)	3.309 (1.097–9.977)	0.034	2.927 (0.954–8.979)	0.060
	Moderate	24 (15.3%)	3.338 (1.116–9.989)	0.031	3.055 (1.002–9.316)	0.048
	Severe	44 (20.0%)	4.624 (1.604–13.336)	0.005	3.881 (1.306–11.534)	0.015
	Linear Trend		/	0.004	/	0.024
NSVT	None	2 (2.6%)	Reference	Reference	Reference	Reference
	Mild	5 (3.4%)	1.357 (0.257–7.162)	0.719	1.360 (0.251–7.370)	0.722
	Moderate	6 (3.8%)	1.510 (0.298–7.659)	0.619	1.326 (0.255–6.897)	0.737
	Severe	24 (10.9%)	4.653 (1.073–20.169)	0.040	3.690 (0.809–16.036)	0.046
	Linear Trend		/	0.001	/	0.013

Adjusted for: age, gender, BMI, smoking, drinking, hypertension, diabetes mellitus, myocardial infarction. AF, atrial fibrillation; NVST, non-sustained ventricular tachycardia; CI, confidence interval; OR, odds ratios.

### Correlation analysis between severe OSA with HRV and ECHO indicators

On the basis of Tables 1, 3, the correlations of severe OSA with significant indicators of HRV and ECHO indicators are shown in Table 5. After full adjustment for confounders, multivariate linear regression model showed that increased LAD, LVDD, RAD and RVD were independently associated with severe OSA (*β* = 1.638, *p* = 0.004; *β* = 1.069, *p* = 0.032; *β* = 1.418, *p* = 0.003; *β* = 0.590, *p* = 0.012, respectively) after full adjustment for confounders. Furthermore, after removing the influence of patients with AF, multivariate analysis demonstrated that severe OSA (*β* = 151.424, *p* = 0.010 and *β* = 0.334; *p* = 0.036, respectively) remained significantly associated with increases in LF and LF/HF.

**Table 5:** Association of OSA (categorized by severity) with differential HRV indices and ECHO indicators.

Dependent variable	Independent variable: severe OSA (unadjusted)			Independent variable: severe OSA (adjusted)		
	*β* (95% CI)	*p*-value	*R*^2^	*β* (95% CI)	*p*-value	R^2^
HRV indicators*						
SDNN (ms)	−5.614 ([−12.443]–1.215)	0.107	0.004	−1.229 ([−8.556]–5.967)	0.725	0.018
RMSSD (ms)	−2.951 ([−6.770]–0.869)	0.130	0.003	−1.811 ([−5.878]–2.255)	0.382	0.029
PNN50 (%)	−1.138 ([−3.015]–0.739)	0.234	0.001	−0.867 ([−2.887]–1.152)	0.399	0.004
LF (ms^2^)	153.666 (47.478–259.854)	0.005	0.016	151.424 (36.904–265.943)	0.010	0.012
LF/HF	0.439 (0.140–0.739)	0.004	0.016	0.334 (0.022–0.647)	0.036	0.076
ECHO indicators						
AAOD (mm)	1.287 (0.650–1.924)	<0.001	0.024	0.618 ([−0.015]–1.250)	0.055	0.143
MPAD (mm)	0.793 (0.290–1.297)	0.002	0.014	0.566 (0.039–1.093)	0.075	0.044
LAD (mm)	2.913 (1.822–4.004)	<0.001	0.043	1.638 (0.537–2.739)	0.004	0.133
LVDD (mm)	2.354 (1.356–3.351)	<0.001	0.034	1.069 (0.090–2.048)	0.032	0.170
RAD (mm)	1.968 (1.055–2.880)	<0.001	0.028	1.418 (0.479–2.357)	0.003	0.088
RVD (mm)	0.819 (0.374–1.264)	<0.001	0.020	0.590 (0.131–1.049)	0.012	0.072
LVPWD (mm)	0.548(0.129–0.968)	0.010	0.009	0.413 ([−0.030]–0.857)	0.068	0.018
LVEF (%)	−1.748 ([−3.021]-[−0.474])	0.007	0.011	−0.972 ([−2.300]–0.356)	0.151	0.047

Adjusted for: age, gender, BMI, smoking, drinking, hypertension, diabetes mellitus, myocardial infarction. *The study population excluded patients with atrial fibrillation. SDNN, standard deviation of normal-to-normal intervals; RMSSD, root mean square successive difference; PNN50, percentage of adjacent NN intervals; LF, power in low frequency spectrum; LF/HF, ratio of low frequency and high frequency; AAOD, ascending aortic diameter; MPAD, main pulmonary artery diameter; LAD, left atrial diameter; LVDD, left ventricular diastolic diameter; RAD, right atrial diameter; RVD, right ventricular diameter; LVPWD, left ventricular posterior wall thickness; LVEF, left ventricular ejection fraction; CI, confidence interval.

### Mediation analysis

**Figure 2:**
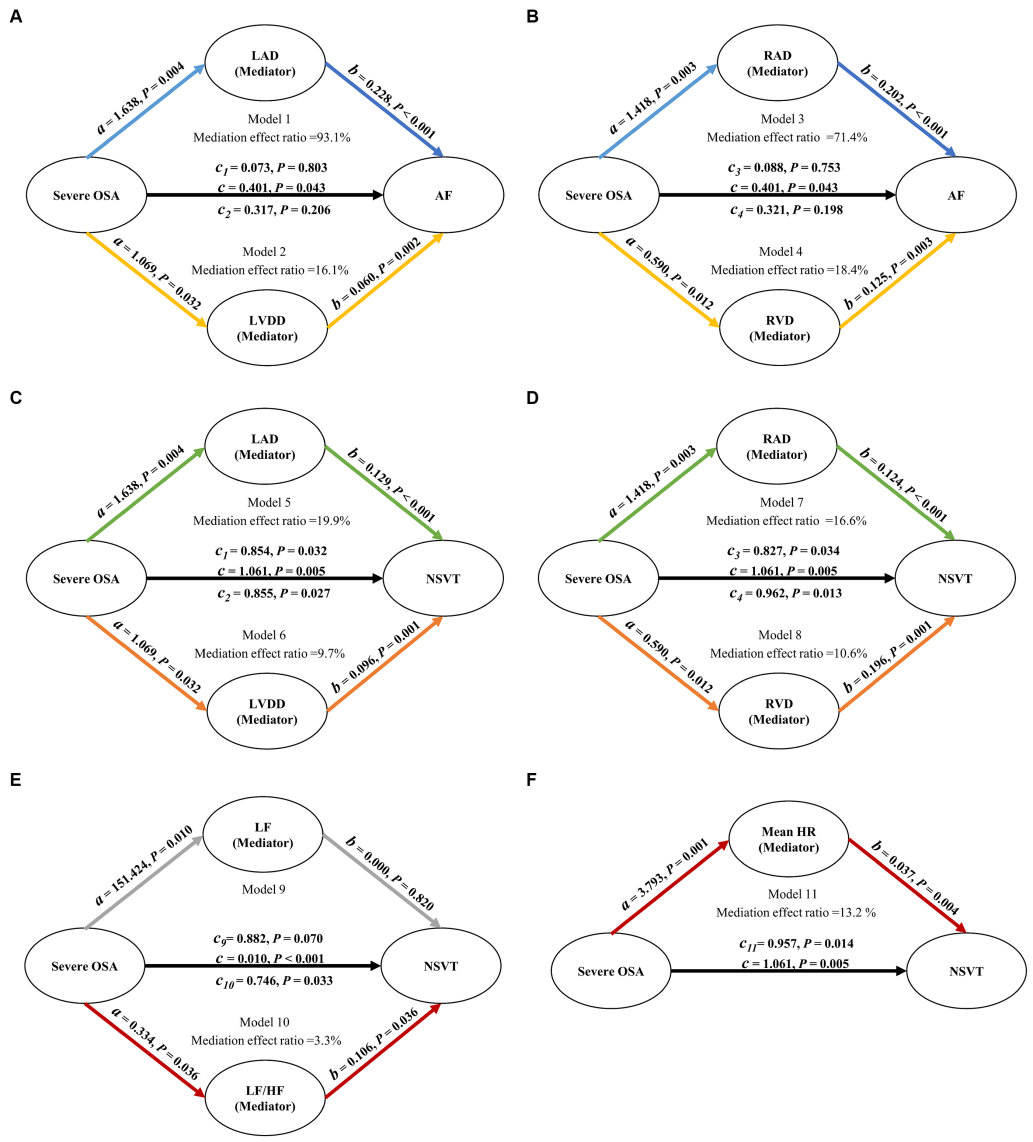
Path diagram of mediation analysis. **(A–D)** LAD, LVDD, RAD, and RVD mediated the relationship between severe OSA and AF. **(C,D)** LAD, LVDD, RAD, and RVD mediated the relationship between severe OSA and NSVT. **(E)** After removing the influence of patients with AF, LF/HF mediated the relationship between severe OSA and NSVT. All analyses were adjusted for age, gender, BMI, smoking, drinking, hypertension, diabetes mellitus and myocardial infarction. OSA, obstructive sleep apnea; LAD, left atrial diameter; LVDD, left ventricular diastolic diameter; RAD, right atrial diameter; RVD, right ventricular diameter; LF, power in low frequency spectrum; LF/HF, ratio of low-frequency and high-frequency; HR, mean heart rate; AF, atrial fibrillation; NSVT, nonsustained ventricular tachycardia.

Based on multivariate linear regression analysis, we examined whether severe OSA and AF and NVST were mediated by the significant HRV and ECHO indicators. Table 6 shows each step of the mediation analysis. These analyses, adjusted for potential confounders, yielded a mediation effect ratio that means how much the mediation effect accounts for the total effect of the independent variable on the dependent variable. In the mediation analysis between severe OSA and AF, the decomposition of the total effect showed that 93.1% of the total effect was mediated by the LAD, 16.1% of the total effect was mediated by the LVDD, 71.4% of the total effect was mediated by the RAD and 18.4% of the total effect was mediated by the RVD. And none of the controlled direct effect (effect directly associated with severe OSA) were significant (all *p* > 0.05). The relationship between severe OSA severity and AF depends almost entirely on LAD, LVDD, RAD and RVD (Figures 2A, B). In the mediation analysis between severe OSA and NSVT, the decomposition of the total effect showed that although the direct effect of severe OSA on NSVT remained significant (all *p* < 0.05), 19.9% of the total effect was mediated by the LAD, 9.7% of the total effect was mediated by the LVDD, 16.6% of the total effect was mediated by the RAD, and 10.6% of the total effect was mediated by the RVD (Figures 2C, D).

**Table 6:** Mediation analysis testing associations of severe OSA with AF and NVST mediated by significant ECHO and HRV indicators.

Model	a	p	b	p	c_n_	p	c	p	Mediation effect ratio (%)
1	1.638	0.004	0.228	<0.001	0.073	0.803	0.401	0.043	93.1
2	1.069	0.032	0.060	0.002	0.317	0.206	0.401	0.043	16.1
3	1.418	0.003	0.202	<0.001	0.088	0.753	0.401	0.043	71.4
4	0.590	0.012	0.125	0.003	0.321	0.198	0.401	0.043	18.4
5	1.638	0.004	0.129	<0.001	0.854	0.032	1.061	0.005	19.9
6	1.069	0.032	0.096	0.001	0.855	0.027	1.061	0.005	9.7
7*	1.418	0.003	0.124	<0.001	0.827	0.034	1.061	0.005	16.6
8*	0.590	0.012	0.196	0.001	0.962	0.013	1.061	0.005	10.9
9	151.44	0.010	0.000	0.820	0.882	0.070	0.010	<0.001	NA
10	0.334	0.036	0.106	0.036	0.746	0.033	0.010	<0.001	3.3

Mediation analysis by multivariate linear regression analysis and multivariate logistic regression, testing associations of severe OSA with AF and NSVT mediated by mean HR, HRV and ECHO indices. All analyses were adjusted for age, gender, BMI, smoking, drinking, hypertension, diabetes mellitus and myocardial infarction. *The study population excluded patients with atrial fibrillation. Model 1–4, Y = AF, X = Severe OSA and M (Mediator) = ECHO indicators (LAD, LVDD, RAD or RVD). Model 2–8, Y = NSVT, X = Severe OSA and M (Mediator) = ECHO indicators (LAD, LVDD, RAD or RVD). Model 9–10, Y = NSVT, X = Severe OSA and M (Mediator) = HRV indicators (LF or LF/HF). Analysis of Y and X = c; Analysis of M and X = a; Analysis of Y and M (together with X) = b; Analysis of Y and X (together with M) = c_n._; Mediation effect ratio = ab/c. NA, not available.

Considering the impact of AF on HRV, we also explored the associations mediated by LF and LF/HF between severe OSA and NSVT in patients who excluded atrial fibrillation. Compared with LF which indirect effect was not significant (*b* = 0.000; *p* = 0.820), the decomposition of the total effect indicated that 3.3% of the total effect was mediated by the LF/HF (Figure 2E).

## Discussion

This study was the first to simultaneously focus on changes in the autonomic nervous system and cardiac structural remodeling in the connection between OSA and arrhythmia, and conveyed several new findings at the same time. First, severe OSA was an independent predictor of AF and NSVT and incident risk of the latter two diseases was augmented with the increase in OSA severity. Second, multiple HRV and ECHO indicators were significant factors associated with severe OSA after adjusting for potential confounders. Finally, we found that the association between severe OSA with AF and NVST was mediated by cardiac structural changes including LAD, LVDD, RAD and RVD. In addition, LF/HF individually mediated the association between severe OSA and NVST.

Many previous reports have shown the relationship between sleep disordered and cardiac arrhythmias in subjects with varying OSA severities, such as conduction delay arrhythmias (13), supraventricular arrhythmias, NVST (8, 14, 15) and AF (8, 16, 17). Both OSA and CSA were thought to predispose to cardiac arrhythmias, but complex ventricular ectopy was most strongly associated with OSA and hypoxia compared to CSA (10). A large multicenter study reported a significantly increased prevalence of AF (4.8% vs. 0.9%) and NSVT (5.3% vs. 1.2%) with severe OSA compared to those without OSA. Upon controlling for age, gender, BMI, and the presence of coronary artery disease, it was observed that those suffering from severe OSA were four times more likely to have AF and three times more likely to have NSVT (8). Another study showed a significant increase in the relative risk of AF and NVST during sleep shortly after respiratory disorders (11). Moreover, the rate of AF recurrence following cardioversion was found to be higher in untreated OSA patients (82%) compared to those with treated OSA (42%) and the control group without OSA (53%) (17). These results support the direct relationship between OSA with AF and NVST. However, it was not clear how this association arose and developed in patients with OSA.

There could be several mechanisms that potentially lead to an increased occurrence of AF and NSVT in patients diagnosed with OSA. On one hand, acute physiological changes resulting from airway collapse during sleep played an important role in arrhythmias caused by OSA. These alterations encompass hypoxemia, hypercapnia, changes in sympathetic and parasympathetic tension, and fluctuations in thoracic pressure (18). On the other hand, the chronic impacts of persistent and recurrent OSA were ultimately linked with cardiac structural remodeling (19, 20). In line with these mechanisms, multivariate linear regression analysis revealed that increased LAD, LVDD, RAD, RVD and LF/HF were associated with severe OSA in this study population. Moreover, our results demonstrated that the association between OSA with AF and NVST was mediated in part through autonomic nervous system changes and cardiac structural remodeling by mediation analysis.

Firstly, prior research has indicated that intermittent hypoxemia, coupled with hypercapnia via chemoreflexes, resulted in heightened sympathetic activation and increased levels of catecholamines in patients with OSA, even during daytime normoxic wakefulness (21). HRV served as a valuable clinical indicator of autonomic balance. In another large-scale study of 4,152 participants to identify the impacts that OSA related to rapid eye movement (REM) exerts on cardiac autonomic dysfunction, measures of HRV revealed a transition to sympathetic predominance in OSA during REM sleep, manifesting as an increased LF/HF and LF (n.u) (22). Tachycardia, resulting from heightened sympathetic activation, led to the consumption of myocardial oxygen at the lowest blood oxygen saturation levels (18). This could potentially cause myocardial ischemia and impair cardiac contractility and diastolic relaxation. We found that increased LF/HF significantly mediated the association between severe OSA and NSVT, and this result was consistent with the previous conclusion.

Second, intrathoracic negative pressure fluctuations during inspiration against a collapsed upper airway in OSA stimulated cardiac mechanoreceptors and increased cardiac transmural pressure (a powerful stimulus to LV hypertrophy), which might mechanically stretch the myocardial walls, thereby promoting significant changes in myocardial excitability and structural remodeling of the myocardium (1, 18, 23). In addition, these forces also lead to a leftward displacement of the interventricular septum during diastole, which hinders LV filling and subsequently decreases stroke volume (12, 24). This repetitive mechanism, occurring during each stage of apnea, might cause stretching of the cardiac wall and intrathoracic vessels. Consequently, it potentially results in both short-term electrical and long-term mechanical remodeling of the atrium and LV, thereby increasing the risk of the occurrence of atrial and ventricular dysrhythmias (12, 24). Chronic LA enlargement and diastolic dysfunction were observed in numerous animal models of chronic OSA (25, 26). Moreover, Neilan et al. (20) reported that patients with OSA had increased pulmonary artery pressure, RA size, LA size, and LV mass compared with those without OSA. Since it was well established that enlarged LAD was an independent risk factor for AF and NVST (14, 27), this fact might help to explain the increased risk of AF and NVST in patients with OSA.

However, the existing literature on conduction delay arrhythmias is limited and often contradictory. Some studies have reported a prevalence of 13.3% in patients with OSA, compared to only 3% in healthy population (1). Conversely, another study found no significant differences in the occurrence of conduction delay arrhythmias between OSA patients and matched control groups (8). Our study results revealed no significant difference in conduction delay arrhythmia between subjects with OSA and those without. This observation may be attributed to variations in study design and the characteristics of the study population.

Finally, increased systemic inflammation, oxidative stress and vascular dysfunction caused by OSA may serve as intermediate pathways for triggering arrhythmias (3, 12). These pathophysiological changes may synergistically exacerbate the occurrence of arrhythmias in patients with OSA.

AF and NSVT were significant risk factors for cardiovascular death and sudden cardiac death in patients with OSA (23, 28). In multiple large prospective cohort trials, the incidence of fatal and non-fatal cardiovascular events was higher in patients with severely untreated OSA compared to patients without OSA. There is a correlation between OSA severity and the risk of cardiovascular disease, however, the effectiveness of CPAP significantly decreases the cardiovascular outcomes related to this disease (29–31). Meanwhile, CPAP could reduce the occurrence of ventricular arrhythmia and reduce the recurrence rate of AF after electrical cardioversion by other studies (17, 20, 32). International professional societies recommended that patients with severe OSA should receive CPAP therapy as early as possible (3). Consistent with this recommendation, we anticipate that prompt diagnosis and timely CPAP therapy will decrease the occurrence of AF and NSVT and potentially lower the frequency of cardiovascular disease mortality. Consequently, further investigations are needed to establish whether treatment of OSA during sleep may reduce the risk of cardiac arrhythmia; such studies include determining the optimal CPAP usage to reverse adverse cardiovascular and metabolic outcomes.

Meanwhile, Sinha et al. (33) reported that cardiac resynchronization therapy might lead to a reduction of CSA and to increased sleep quality in patients with heart failure and SDB. In addition, in another prospective study of 67 patients with mitral regurgitation, patients with mitral regurgitation and SDB who underwent MitraClip-placement showed a significant AHI improvement (34). Although the study sample is small and the specific mechanism still needs to be studied, this provides new ideas for the treatment of OSA.

The present study has the following limitations. First, due to the relatively small number of cases of NVST in each group, the OR confidence intervals for cardiac arrhythmias in patients with severe OSA were wide, despite adequate test reliability. Second, data on cardiac arrhythmias and OSA in the context of mortality are scarce and all additional information can help to create a more complete picture. Lastly, the study was the lack of follow-up of patients with diagnosed OSA-related cardiac arrhythmias who were eligible for CPAP therapy. Therefore, further work is needed to determine the prognostic significance of CPAP in NSVT and AF.

## Conclusion

This study demonstrated that severe OSA was independently associated with AF and NSVT, and this association was mediated by autonomic nervous system changes and cardiac structural remodeling. Further research is required to clarify the exact mechanisms underlying the relationship of OSA with AF and NSVT, and to assess the effect of OSA therapy on the development, treatment, and prognosis of AF and NSVT.

## Data availability statement

The original contributions presented in the study are included in the article/supplementary material, further inquiries can be directed to the corresponding author.

## Ethics statement

The studies involving humans were approved by ethics committee of Renmin Hospital of Wuhan University. The studies were conducted in accordance with the local legislation and institutional requirements. The participants provided their written informed consent to participate in this study. Written informed consent was obtained from the individual(s) for the publication of any potentially identifiable images or data included in this article.

## Author contributions

HC: Methodology, Writing – original draft. QZ: Methodology, Writing – original draft. YuH: Data curation, Writing – original draft. JZ: Software, Writing – original draft. YaH: Supervision, Visualization, Writing – original draft. KH: Funding acquisition, Resources, Writing – review & editing.
